# Clinical Prediction Models Incorporating Blood Test Trend for Cancer Detection: Systematic Review, Meta-Analysis, and Critical Appraisal

**DOI:** 10.2196/70275

**Published:** 2025-06-27

**Authors:** Pradeep S Virdee, Kiana K Collins, Claire Friedemann Smith, Xin Yang, Sufen Zhu, Nia Roberts, Jason L Oke, Clare Bankhead, Rafael Perera, FD Richard Hobbs, Brian D Nicholson

**Affiliations:** 1 Nuffield Department of Primary Care Health Sciences, University of Oxford, Radcliffe Primary Care Building, Woodstock Road, Oxford, OX2 6GG, United Kingdom, 44 1865617855; 2 St Edmund Hall, University of Oxford, Oxford, United Kingdom; 3 Bodleian Health Care Libraries, University of Oxford, Oxford, United Kingdom

**Keywords:** blood test, hematologic tests, trend, prediction model, primary health care, cancer, neoplasms, systematic review

## Abstract

**Background:**

Blood tests used to identify patients at increased risk of undiagnosed cancer are commonly used in isolation, primarily by monitoring whether results fall outside the normal range. Some prediction models incorporate changes over repeated blood tests (or trends) to improve individualized cancer risk identification, as relevant trends may be confined within the normal range.

**Objective:**

Our aim was to critically appraise existing diagnostic prediction models incorporating blood test trends for the risk of cancer.

**Methods:**

MEDLINE and EMBASE were searched until April 3, 2025 for diagnostic prediction model studies using blood test trends for cancer risk. Screening was performed by 4 reviewers. Data extraction for each article was performed by 2 reviewers independently. To critically appraise models, we narratively synthesized studies, including model building and validation strategies, model reporting, and the added value of blood test trends. We also reviewed the performance measures of each model, including discrimination and calibration. We performed a random-effects meta-analysis of the c-statistic for a trends-based prediction model if there were at least 3 studies validating the model. The risk of bias was assessed using the PROBAST (prediction model risk of bias assessment tool).

**Results:**

We included 16 articles, with a total of 7 models developed and 14 external validation studies. In the 7 models derived, full blood count (FBC) trends were most commonly used (86%, n=7 models). Cancers modeled were colorectal (43%, n=3), gastro-intestinal (29%, n=2), nonsmall cell lung (14%, n=1), and pancreatic (14%, n=1). In total, 2 models used statistical logistic regression, 2 used joint modeling, and 1 each used XGBoost, decision trees, and random forests. The number of blood test trends included in the models ranged from 1 to 26. A total of 2 of 4 models were reported with the full set of coefficients needed to predict risk, with the remaining excluding at least one coefficient from their article or were not publicly accessible. The c-statistic ranged 0.69‐0.87 among validation studies. The ColonFlag model using trends in the FBC was commonly externally validated, with a pooled c-statistic=0.81 (95% CI 0.77-0.85; n=4 studies) for 6-month colorectal cancer risk. Models were often inadequately tested, with only one external validation study assessing model calibration. All 16 studies scored a low risk of bias regarding predictor and outcome details. All but one study scored a high risk of bias in the analysis domain, with most studies often removing patients with missing data from analysis or not adjusting the derived model for overfitting.

**Conclusions:**

Our review highlights that blood test trends may inform further investigation for cancer. However, models were not available for most cancer sites, were rarely externally validated, and rarely assessed calibration when they were externally validated.

## Introduction

Cancer incidence trends are projected to increase globally: 18 million new cases diagnosed in 2020 versus 28 million projected in 2040 [1]. The likelihood of survival improves by cancer detection at earlier stages [2-7]. Earlier detection is crucial to improve patient outcomes and reduce cancer-related mortality [8]. Screening programs may contribute to early detection but have been implemented for a minority of countries and cancers [9]. Risk prediction models for cancer could improve early detection rates. These models combine patient data, such as patient demographics, medical history, or cancer symptoms, to identify patients with an increased risk of undiagnosed cancer.

Blood tests commonly performed in clinical practice, including full blood count (FBC) and liver function tests, are often included in cancer risk prediction models, as they have an important role in risk-stratifying symptomatic patients for cancer investigation [10,11]. Blood tests are commonly requested by clinicians, with rates of testing increasing yearly. Despite panels of blood tests being taken together, blood tests are almost entirely interpreted in isolation in current clinical guidance [11,12]. In the United Kingdom, the National Institute for Health and Care Excellence (NICE) suspected cancer guidelines recommend referral for urgent investigation if low albumin, low hemoglobin, raised platelets, raised bilirubin, raised calcium, or raised inflammatory markers are observed, as these increase risk of cancer [11]. Monitoring temporal trends (ie, changes over time) in repeated blood tests may improve risk stratification, by incorporating an individual’s trajectory from which to identify change. For example, declining hemoglobin confined within the normal range would be a relevant cancer-related trend, but missed in practice as the results appear normal. Our recent systematic review on the association between blood test trends and cancer diagnosis identified many trends that have the potential to improve cancer risk stratification [13]. However, the potential benefits and challenges and methodological considerations of incorporating combinations of trends into cancer risk prediction models remain unrealized.

Recent methodological advancements in both traditional statistical and machine-learning methods allow for the development of dynamic prediction models, which incorporate repeated measures data for clinical risk prediction and may hold greater potential to rule-in and rule-out referral for cancer investigation. We aimed to conduct a systematic review to critically appraise diagnostic clinical prediction models using trends in blood tests commonly used in primary care for the risk of undiagnosed cancer.

## Methods

### Overview

We followed the PRISMA (Preferred Reporting Items for Systematic review and Meta-Analysis) guidelines (Checklist 1) for reporting the findings of this review [14]. Ethical approval was not required, as there were no direct patient investigations in this study and only published articles were systematically reviewed. The review protocol was registered with the International PROSPERO (Prospective Register of Systematic Reviews) database on July 25, 2022 (CRD42022348907). There were no deviations to the protocol.

### Participants

We included studies of participants aged 18 years or older reporting prediction models incorporating trends in blood tests commonly available in primary care and cancer diagnosis in any clinical setting. We excluded blood tests taken after cancer diagnosis, such as to predict prognosis or monitor treatment.

### Outcome

The main outcome was a first diagnosis of cancer across all cancer sites, including composite cancer sub-groupings and all cancers combined. Cancer diagnosis was defined as per the individual studies, such as confirmed cancer via laboratory tests/radiology in clinical/prospective studies or the use of ICD10 (*International Statistical Classification of Diseases and Related Health Problems 10th Revision)* codes [15] in studies of eHealth records.

### Search Strategy

We worked with our review specialist (NR) to derive a comprehensive search strategy. The MEDLINE (OVID) (1946-present) and EMBASE (OVID) (1974-present) databases were searched from inception to April 3, 2025 to identify articles that report on the association between trends in blood tests commonly available in clinical practice and a cancer diagnosis. The initial search was conducted in June 2022, with a full update in February and May 2023 and April 2025. Search terms included MeSH headings and title, abstract, and author keywords for blood tests, cancer diagnosis, and prediction or risk. Cancer-related terms included “tumor” and “cancer”. However, some cancers are not usually paired with these terms, such as “leukaemia” or “lymphoma”, so it was important to include such cancer types explicitly to ensure they were captured. No language or other limits were applied to the search. The full search strategy for each database is provided in Table S1 (MEDLINE) and Table S2 (EMBASE) in Multimedia Appendix 1. In the eligible studies, we actively searched through each article’s reference list to find eligible studies that were not identified by the search strategy.

### Study Selection

All references initially underwent de-duplication in Endnote 20 [16] (by NR). Abstract and title screening was performed in Endnote 20 and Rayyan [17] (by PSV, KKC, CFS, and XY). The retrieved articles were initially split among the reviewers for screening, with a sample of 1000 from each of the three reviewers (KKC, CFS, and XY) independently screened by a second reviewer (PSV) to assess agreement, with discrepancies discussed until an agreement was reached. The full-text screening was subsequently performed independently by two reviewers (by PSV and SZ) to identify eligible articles for data extraction and analysis, with discrepancies discussed until agreement was reached. We included any in-human primary research article reporting the development or validation of a diagnostic clinical risk prediction model using a prediagnostic trend over repeat measurements of at least one blood test parameter (Table 1) for subsequent diagnosis of cancer. A prediction model was defined as any multivariable model designed to predict the presence of undiagnosed cancer (outcome), where at least one predictor in the model was a blood test trend. A model was considered to include “trend” if it included temporal changes in the quantitative blood test result over repeatedly measured tests per patient as a predictor. The blood tests in Table 1 are nonspecific (ie, not cancer-specific) blood tests that are commonly available in primary care settings. Recent evidence highlighted trends in many of these common tests as risk factors for cancer diagnosis [13]. Using these blood tests provides an opportunity to use commonly available data to support cancer detection.

**Table 1.** Blood tests included in this review.

Blood test	Blood level
Full blood count	Red blood cell count, hemoglobin, hematocrit, mean cell volume, mean cell hemoglobin, mean cell hemoglobin concentration, red blood cell distribution width, platelet count, mean platelet volume, white blood cell count, basophil count, eosinophil count, lymphocyte count, monocyte count, neutrophil count, basophil %, eosinophil %, lymphocyte %, monocyte %, neutrophil %
Liver function tests	Alanine aminotransaminase, albumin, alkaline phosphatase, aspartate transaminase, bilirubin
Renal function	Sodium, potassium, creatinine, urea
Inflammatory markers	C-reactive protein, erythrocyte sedimentation rate, plasma viscosity
Other tests	Amylase, HbA_1c^a^_, calcium, calcium adjusted, total protein, blood glucose, fasting glucose, thyroid stimulating hormone

a HbA_1c_: hemoglobin A1c.

We excluded abstracts and conference proceedings, as they produce incomplete data for a thorough review. Studies using a cross-sectional design were excluded, as the data reflects a “snapshot” at a certain time so cannot assess risk over time. Clinical trials of treatment intervention were excluded to reduce the influence of treatments on blood test data. Existing systematic reviews, correspondence, and case studies pertaining to<5 individuals were excluded. Non-English full-texts without English versions available or nontranslatable were excluded.

### Data Extraction

Data was extracted using an extraction form designed in Microsoft Excel and piloted on 3 randomly selected eligible articles. Data items included study design and population, blood test trends studied, analytic methods, cancer site, and predictive performance measures. Data extraction from each eligible article was performed by 2 reviewers independently (PSV, KKC, CFS, XY, and SZ), with disagreements discussed until agreement was reached.

### Data Analysis and Synthesis

Quantitative data were summarized using means with SD for continuous data and counts with proportions for categorical data. We narratively described and critically appraised prediction models incorporating prediagnostic blood test trend. We performed a random-effects meta-analysis of the c-statistic (or area under the curve) for prediction models externally validated by at least 3 studies. The τ^2^ statistic was used to describe heterogeneity and *I*^2^ statistic to assess the proportion of heterogeneity explained by between-study differences. We also conducted a post hoc analysis, repeating the meta-analysis by including only studies using primary care data and again using only other studies, to assess if findings differed between underlying populations of care. Analyses were performed in Stata/SE 17.0.

### Risk of Bias Assessment

Risk of bias in each study was assessed using the Cochrane Prediction model Risk Of Bias Assessment Tool (PROBAST) [18]. Each study was assessed by two reviewers independently (PSV, KKC, CFS, XY, and SZ), with disagreements discussed until agreement was reached. Articles coauthored by a reviewer were assessed by other reviewers.

## Results

### Overall Summary

**Figure 1.**
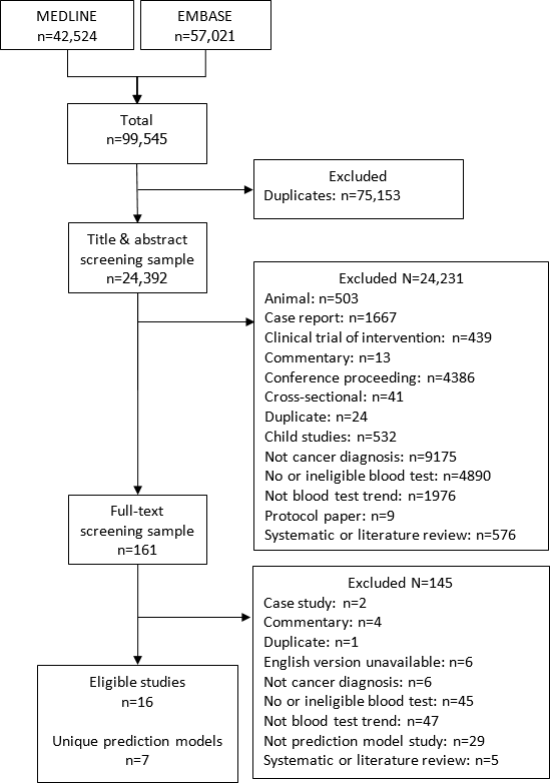
PRISMA (preferred reporting items for systematic review and meta-analysis) diagram.

In total, 99,545 references were identified, of which 24,392 were unique after deduplication (Figure 1). A total of 16 studies met the eligibility criteria and were included in the review [19-34]. A total of 7 blood test trend-based prediction models were developed in total among 5 studies [23,27,28,30,31] and the remaining 11 studies [19-22,24-26,29,32-34] externally validated existing prediction models. In total, there were 14 external validations of 2 models (ColonFlag by Kinar et al [27] and ENDPAC (Enriching New-Onset Diabetes for Pancreatic Cancer) by Sharma et al [30]).

### Description of Studies

#### Study Design

A description of each study is provided in Table S3 in Multimedia Appendix 1. Of the 16 studies, a case-control design was used by 19% (n=3) [23,25,29] and cohort design by 81% (n=13) [19-22,24,26-28,30-34]. In addition, 25% (n=4) [19,20,22,24] used prospectively-collected data and 75% (n=12) [21,23,25-34] used retrospective data. Furthermore, 19% (n=3) [19,20,28] collected data at clinical centers, 75% (n=12) [21-23,25-27,29-34] used eHealth record databases, and 6% (n=1) [24] used both. All studies used opportunistic tests (ie, performed for any reason excluding screening for cancer, such as to monitor symptoms or comorbidity).

#### Participants

The mean number of participants recruited was 23,896 among prospective studies and 502,730 among retrospective studies, ranging from 617 to 2,914,589 participants over all the studies. The 16 articles spanned 4 different countries: the United States of America (44%, n=7) [23,25,28-30,33,34], the United Kingdom (25%, n=4) [19-21,31], Israel (25%, n=4) [22,26,27,32], and Canada (6%, n=1) [24]. The period of recruitment ranged from 1996 to 2020 in all studies. There were 38% (n=6) [21,26-28,31,32] studies conducted in primary care, 12% (n=2) [19,20] in secondary care, and 31% (n=5) in other settings: community-based insured adults (n=1) [25], endoscopy unit (n=1) [24], and insured individuals (n=3) [23,29,33]. It was unclear in 18% (n=3) [22,30,34]. One study [24] (6%) was limited to asymptomatic patients, including only patients without symptoms, and the remaining 94% (n=15) [19-23,25-34] included participants regardless of whether they experienced symptoms or not. A total of 6 studies [20,21,24,26,28,31] reported age, with a mean age 58.1 years (SD 5.2) among them. A total of 7 studies [21,25,27-29,31,32] reported sex, with mean 54.9% (SD 3.9) of females among them.

### Model Building Strategy

Characteristics of the 7 models are in Table 2. A total of 4 models (57%) were developed in the USA population [23,28,30], 2 (29%) in United Kingdom [31], and 1 (14%) in Israel [27]. A total of 3 models (43%) were developed for risk of colorectal cancer [27,31], 2 (29%) for gastro-intestinal cancer (defined by Read as cancer of the esophagus, stomach, small intestine, colon, rectum, or anus) [28], 1 (14%) for nonsmall cell lung cancer [23], and 1 (14%) for pancreatic cancer [30]. A total of 6 models assessed cancer risk from the time of the latest blood test included and it was unclear in one study [23].

**Table 2.** Characteristics of 7 trend-based prediction models for cancer diagnosis.

Article	Country	Model (name, if assigned)	Outcome	Outcome risk window	Patient setting	Blood level(s) trend	Number of cases/total	Predictors in the final model
Gould et al [23]	United States of America	MES	Nonsmall cell lung cancer	Diagnosis	Other – insured individuals	ALT^a^, creatinine, blood glucose, MCHC^b^, platelets, RDW^c^, WBC^d^	3942/117669	Age, sex, education, race, marital status, smoking status, smoking pack year, smoking years, smoking intensity, days since quitting, Hospitalization due to COPD and allied conditions, Diagnosis of COPD and allied conditions, Hospitalization due to Cancer, Diagnosis of Cancer, ALT, Creatinine, Glucose, MCHC, Platelets, RDW, WBC
Kinar et al [27]	Israel	ColonFlag	Colorectal cancer	3‐6 months	Primary care	RBC^e^, hemoglobin, hematocrit, MCV^f^, MCH^g^, MCHC, RDW, platelets, MPV^h^, WBC, basophil#, basophil%, eosinophil#, eosinophil%, lymphocyte#, lymphocyte %, monocyte#, monocyte %, neutrophil#, neutrophil %	2437/466107	RBC, hemoglobin, hematocrit, MCV, MCH, MCHC, RDW, platelets, MPV, WBC, basophil#, basophil%, eosinophil#, eosinophil%, lymphocyte#, lymphocyte %, monocyte#, monocyte %, neutrophil#, neutrophil %, age, sex
Read et al [28]	United States of America	Logistic model	Gastrointestinal cancer (esophagus, stomach, small intestine, colon, rectum, or anus)	6 months	Primary care	RBC, hemoglobin, hematocrit, MCV, MCH, MCHC, RDW, platelets, MPV, WBC, basophil#, basophil%, eosinophil#, eosinophil%, lymphocyte#, lymphocyte %, monocyte#, monocyte %, neutrophil#, neutrophil %	1025/148158	Age, sex, race, BMI, RBC, hemoglobin, hematocrit, MCV, MCH, MCHC, RDW, platelets, MPV, WBC, basophil#, basophil%, eosinophil#, eosinophil%, lymphocyte#, lymphocyte %, monocyte#, monocyte %, neutrophil#, neutrophil %, most recent BMP (8 components)
Read et al [28]	United States of America	Machine learning model	Gastrointestinal cancer (esophagus, stomach, small intestine, colon, rectum, or anus)	6 months	Primary care	RBC, hemoglobin, hematocrit, MCV, MCH, MCHC, RDW, platelets, MPV, WBC, basophil#, basophil%, eosinophil#, eosinophil%, lymphocyte#, lymphocyte %, monocyte#, monocyte %, neutrophil#, neutrophil %	1025/148158	Age, sex, race, BMI, RBC, hemoglobin, hematocrit, MCV, MCH, MCHC, RDW, platelets, MPV, WBC, basophil#, basophil%, eosinophil#, eosinophil%, lymphocyte#, lymphocyte %, monocyte#, monocyte %, neutrophil#, neutrophil %, most recent BMP (8 components)
Sharma et al [30]	United States of America	ENDPAC^i^	Pancreatic cancer	3 years	Unclear	Blood glucose	16/256	Change in weight, change in blood glucose category, age, change in blood glucose
Virdee et al [31]	United Kingdom	BLOODTRACC^j^ Colorectal (females)	Colorectal cancer	2 years	Primary care	Hemoglobin, MCV, platelets	677/246695	Age, hemoglobin trend, MCV trend, platelets trend
Virdee [31]	United Kingdom	BLOODTRACC Colorectal (males)	Colorectal cancer	2 years	Primary care	Hemoglobin, MCV, platelets	865/250716	Age, hemoglobin trend, MCV trend, platelets trend

a ALT: alanine aminotransaminase.

b MCHC: mean cell hemoglobin concentration.

c RDW: red blood cell distribution width.

d WBC: white blood cell count.

e RBC: red blood cell count.

f MCV: mean cell volume.

g MCH: mean cell hemoglobin.

h MPV: mean platelet volume.

i ENDPAC: enriching new-onset diabetes for pancreatic cancer.

j BLOODTRACC: full blood count trends for colorectal cancer detection.

In total, 2 models were developed using multivariate joint modeling [31], 2 using logistic regression [28,30], and 1 using each of XGBoost [23], decision trees [27], and random forests [28]. A total of 3 models (43%) were built by including all candidate predictors [27,28], 2 (29%) included clinically relevant predictors that were commonly available in practice [31], 1 (14%) included statistically significant variables in univariable analysis [30], and the model building process was unclear for 1 (14%) model [23]. To address missing blood test data, 2 (29%) models derived missing blood levels from other available blood levels using known mathematical relationships (eg mean cell hemoglobin=hemoglobin/red blood cell count) [31], 2 (29%) used imputation methods [28], 1 (14%) analyzed the blood test data as-is (without altering missing data) [23], and 1 (14%) used other methods (linear models to replace missing values using historical blood tests or mean value across all blood tests if no historic blood tests were present) [27]. Methods for handling missing blood test data were not discussed in 1 (14%) study [30].

### Modeling Blood Test Trends

A total of 3 models (43%) assessed trends over repeated quantitative blood test results; Kinar et al [27] used ensembles of decision trees for the ColonFlag model, modeling changes over tests measured at 3‐6 months before diagnosis and 18 and 36 months before that for each patient in the ensemble model, and Virdee et al [31] used multivariate joint modeling, which uses mixed-effects modeling to account for differing numbers of tests and the time between them in sporadically available repeated measures data between patients, for both BLOODTRACC models. One model (14%), by Sharma et al[30], calculated the difference between tests and included this as a single continuous variable in a logistic regression model to determine risk. It was unclear how trends were included in 3 (43%) models to predict risk [23,28].

The number of repeat blood tests used to define trend varies between models. Read et al [28] calculated the change in slope (reflecting the trend/trajectory) over at least 2 repeated tests sporadically measured over 3 years, Sharma et al [30] calculated the difference between blood tests measured at 18-3 months before new-onset diabetes and included this in their model, and Virdee et al[35] included the change in slope across all available blood tests (median=3 per patient) sporadically measured over 5 years to predict risk. The number of repeated blood tests used to derive trends was not reported for 3 models (43%) but the period of repeated testing among them ranged between 18 months and 5 years [23,27,30]. See Table S4 in Multimedia Appendix 1 for further details.

A total of 6 models (86%) used combinations of blood test trends and 1 model (14%) used trend in a single blood test (plus with other patient data) to predict cancer risk. The logistic model and random forests model by Read et al [28] combined trends in 28 blood tests Kinar et al [27]. combined trends in 20 blood tests (that make up the FBC) using decision trees, and Gould et al [23] combined trends in 7 blood tests using XGBoost. Virdee et al [35] combined 3 blood test trends (hemoglobin, mean corpuscular volume, and platelets) using multivariate joint modeing.

### Model Reporting

Total 3 (43%) models were reported using appropriate reporting guidelines to report model findings (TRIPOD [Transparent Reporting of a multivariable prediction model for Individual Prognosis Or Diagnosis] guidelines [28,31,36]). For 3 (43%) models, justification for their choice of outcome risk window was provided [23,31]. In addition, 2 (29%) models were reported to be sufficiently powered, having provided a sample size calculation to show the number of patients and events needed to ensure reliable predictions and minimize optimistic performance [31].

Read et al [28] did not report the coefficients from their logistic model and Sharma et al [30] did not report the intercept from their logistic model. The full risk equation needed to derive an individual’s risk of diagnosis was only reported for 2 models [31]. The models developed using XGBoost, decision trees, and random forests were not reported, due to the nature of machine learning, and a reference to publicly available models was not provided [23,27,28].

### Internal Validation

A total of 6 (86%) models underwent internal validation and one (14%) (by Sharma [30]) did not (Table 3). The internal validation sample was obtained using random data splitting for 4 (57%) models [23,27,31] and cross-validation for 2 (29%) models [23,28]. On average, there were 214,883 participants in the validation samples, ranging from 78,433 to 462,900. A total of 4 (57%) models were adjusted for overestimated performance [27,28,31] and it was unclear for 2 (29%) models [23,28].

**Table 3.** Performance statistics from internal and external validations of the final models, which include trends and other patient data.

Article	Model name/description	Outcome risk window	Overall performance		Discrimination		Calibration	
			Method	Result	Method	Result (95% CI)	Method	Result
Internal validation								
Gould et al [23]	MES	3‐6 months	No		AUC/C-statistic	0.870 (0.856‐0.886)	Isotonic regression	
Gould et al [23]	MES	6‐9 months	No		AUC/C-statistic	0.862 (0.845‐0.878)	No	
Gould et al [23]	MES	9‐12 months	No		AUC/C-statistic	0.856 (0.840‐0.872)	No	
Kinar et al [27]	ColonFlag	1 month	No		AUC/C-statistic	0.84	No	
Kinar et al [27]	ColonFlag	3‐6 months	No		AUC/C-statistic	0.82	Hosmer-Lemeshow test	*P*=.47
Read et al [28]	Logistic regression	6 months	Brier score	0.008	AUC/C-statistic	0.711 (0.691- 0.731)	No	
Read et al [28]	Machine-learning (random forest)	6 months	Brier score	0.092	AUC/C-statistic	0.713 (0.689- 0.737)	No	
Virdee et al [35]	BLOODTRACC^a^ Colorectal (females)	2 years	Brier score	0.0028	AUC/C-statistic	0.763 (0.753‐0.775)	Calibration slope	1.05
Virdee et al [35]	BLOODTRACC Colorectal (males)	2 years	Brier score	0.0033	AUC/C-statistic	0.751 (0.739‐0.764)	Calibration slope	1.06
External validation								
Ayling et al [19]	ColonFlag	Diagnosis	No		No		No	
Ayling et al [20]	ColonFlag	6 months	No		No		No	
Birks et al [21]	ColonFlag	3‐6 months	No		AUC/C-statistic	0.844 (0.839‐0.849)	No	
Birks et al [21]	ColonFlag	6‐12 months	No		AUC/C-statistic	0.813 (0.809‐0.818)	No	
Birks et al [21]	ColonFlag	12‐24 months	No		AUC/C-statistic	0.791 (0.786‐0.796)	No	
Birks et al [21]	ColonFlag	18‐24 months	No		AUC/C-statistic	0.776 (0.771‐0.781)	No	
Birks et al [21]	ColonFlag	24‐36 months	No		AUC/C-statistic	0.751 (0.746‐0.756)	No	
Goshen et al [22]	ColonFlag	Diagnosis	No		No		No	
Hilsden et al [24]	ColonFlag	1 year	No		No		No	
Hornbrook et al [25]	ColonFlag	6 months	No		AUC/C-statistic	0.80 (0.79‐0.82)	No	
Kinar et al [27]	ColonFlag	1 month	No		AUC/C-statistic	0.84 (0.82‐0.86)	No	
Kinar et al [27]	ColonFlag	3‐6 months	No		AUC/C-statistic	0.81 (0.80‐0.83)	Hosmer-Lemeshow test	*P<*.001
Kinar et al [26]	ColonFlag	12‐18 months	No		No		No	
Schneider et al [29]	ColonFlag	6 months	No		AUC/C-statistic	0.78 (0.77‐0.78)	No	
Virdee et al [31](Females)	ColonFlag	2 years	No		AUC/C-statistic	0.761 (0.744‐0.768)	No	
Virdee et al [31] (Males)	ColonFlag	2 years	No		AUC/C-statistic	0.762 (0.749‐0.774)	No	
Boursi et al [32]	ENDPAC^b^	3 years	No		AUC/C-statistic	0.69	No	
Chen et al [33]	ENDPAC	3 years	No		AUC/C-statistic	0.75	No	
Khan et al [34]	ENDPAC	4 years	No		AUC/C-statistic	0.72	No	
[30] Sharma et al [30]	ENDPAC	Diagnosis	No		No		No	

a BLOODTRACC: Full blood count trends for colorectal cancer detection.

b ENDPAC: enriching new-onset diabetes for pancreatic cancer.

Only 4 (57%) models assessed overall performance. Virdee et al [31], derived Brier scores of 0.0028 (men) and 0.0033 (women) for 2-year risk of colorectal cancer and Read et al [28] derived Brier scores of 0.008 (logistic regression) and 0.092 (random forests) for 6-month risk of GI cancer^28^.

A total of 6 (86%) models (100% of those internally validated) assessed discrimination, each using the c-statistic. Gould 2021 [23] and Kinar 2016 [27] reported c-statistic=0.87 and 0.82 for 3‐6-month risk of nonsmall cell lung cancer in the United States of America and Israel based on various blood test trends measured over 5 years combined with other patient data and colorectal cancer based on all FBC parameters over 3 years combined with other patient data, respectively. Read 2023 [28] reported c-statistic=0.711 (logistic regression) and 0.713 (random forests) for 6-month risk of GI cancer based on FBC trends combined with other patient data. Virdee et al [31] reported c-statistic=0.75 (men) and 0.76 (women) for 2-year risk of colorectal cancer following trends in hemoglobin, mean cell volume, and platelets, together with age, measured over 5 years in UK primary care patients.

A total of 4 (57%) models were assessed for calibration. Gould 2021 [23] used isotonic regression to assess calibration, but did not report the corresponding results. Kinar 2016 [27] used the Hosmer-Lemeshow test and reported *P*=.47 for 3‐6 month risk of colorectal cancer. Virdee et al [31] derived calibration slopes of 1.06 (men) and 1.05 (women) for 2-year risk of colorectal cancer and presented calibration plots.

### External Validation

Fourteen external validation studies were performed in total for 2 models (Table 3): the ColonFlag by [27] was externally validated by 10 studies and the ENDPAC model by [30] by 4 studies. There were on average 244,580 participants included in the external validation studies, ranging from 532 to 2,225,249. Overall performance, discrimination, and calibration are all essential assessments to assess external validity of prediction models [37]. Overall performance of the ColonFlag or ENDPAC model was not assessed during external validation.

A total of 6 (29%) of the 14 external validations assessed discrimination, with all using the c-statistic. Birks et al [21] externally validated ColonFlag at multiple time intervals between the most recent blood test and diagnosis in a UK sample, reporting c-statistic=0.844 at 3‐6 months, which reduced to 0.751 at 23‐36 months [21]. Kinar et al [27] also externally validated the ColonFlag using UK data and reported a similar c-statistic (0.81) at 3‐6 months before colorectal cancer diagnosis [27]. However, Kinar et al [27] removed the red blood cell distribution width blood level from the model and assessed predictive performance of the resulting model. This was because the UK dataset did not include red blood cell distribution width, but the removal of a predictor from the model consequently means the external validation is incomplete.

**Figure 2.**
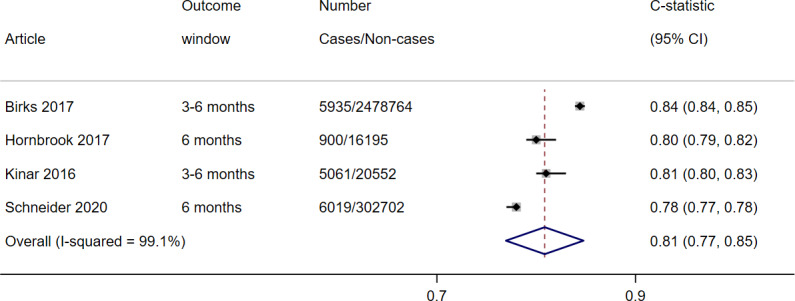
Forest plot of c-statistic for risk of colorectal cancer from ColonFlag external validations [21,25,27,29].

A total of 4 studies with available data assessed <6-month risk of colorectal from ColonFlag and were included in a random-effects meta-analysis [21,25,27,29]. The pooled estimate indicated c-statistic=0.81 (95% CI 0.77‐0.85) (τ^2^=0.0016), with 99.1% (*I*^2^) of the heterogeneity attributable to between-study differences (Figure 2). Our post hoc meta-analyses including only primary care populations and nonprimary care populations separately reduced heterogeneity, but this remained high (Figure S1 in Multimedia Appendix 1).

Calibration was assessed by Kinar et al [27]2016 only, using the Hosmer-Lemeshow test for the ColonFlag. They reported weak calibration at 3‐6 months in the UK dataset (*P*<.001).

### Added Value of Trend

Kinar et al [27] assessed which blood test trends contributed most to the c-statistic of their prediction model for 3‐6 month risk of colorectal cancer. Their model included trend in 20 FBC parameters, age, and sex. Red blood cell-related parameters contributed the most to the c-statistic, with trend in hemoglobin contributing the most (around 0.11) when added to age and sex. White blood cell-related parameters added the least to the c-statistic when combined with age and sex, such as adding around 0.03 AUC with the inclusion of monocyte count trend.

Read et al [28] used logistic regression to develop prediction models for the 6-month risk of gastro-intestinal cancer, including age, sex, BMI, blood test trends, and further covariates. They compared the c-statistic of their final model to one including blood tests measured at a single time point (the last test prior to the prediction interval). They report a higher c-statistic for their model including blood test trends (0.711, 95% CI 0.691‐0.731) compared with the model including blood tests from a single time point (0.697, 95% CI 0.679‐0.715). As secondary analyses, they assessed the c-statistic for one-, three-, and five-year risk, reporting higher c-statistics for models including blood test trends compared to models including single blood tests for one- (0.705, 95% CI 0.689‐0.722 trend and 0.693, 95% CI 0.675‐0.710 single) and three-year (0.735, 95% CI 0.713‐0.757 trend and 0.683, 95% CI 0.665‐0.701 single) risk but a lower c-statistic for their model including trends for five-year risk (0.672, 95% CI 0.653‐0.691 trend and 0.703, 95% CI 0.686‐0.720 single). No other study reported the added benefit of blood test trend to the prediction models.

### Risk of Bias

**Figure 3.**
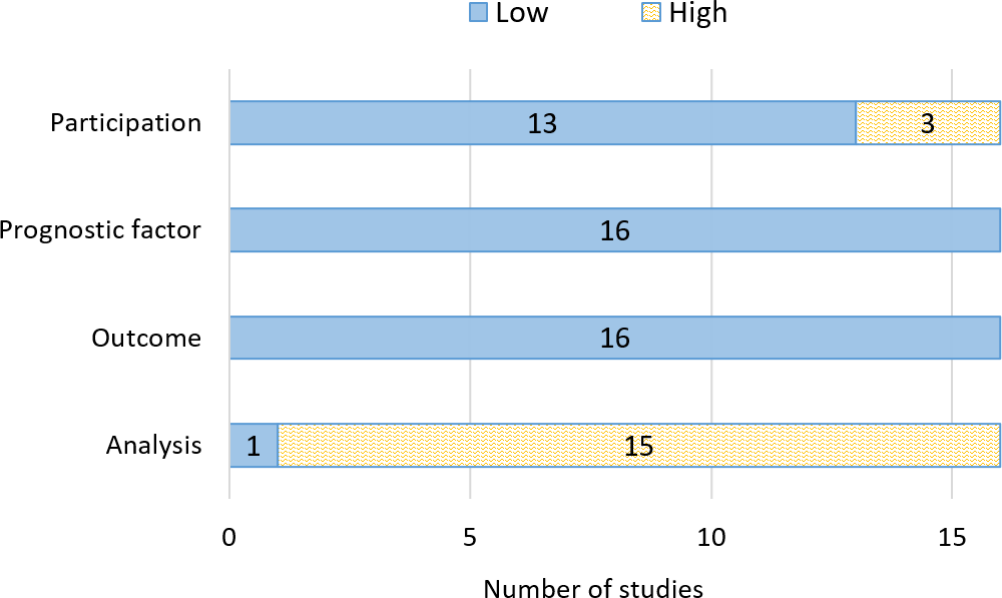
Summary of risk of bias scores, assessed using the prediction model risk of bias assessment tool.

Risk of bias for each domain is summarised in Figure 3 and per study in Table S5 in Multimedia Appendix 1. All 16 studies scored a low risk of bias in the predictors and outcome domains. All but 3 studies in the participant domain scored low risk of bias, with (Gould et al, Hornbrook et al, and, Schneider et al [23,25,29]) scoring high risk of bias for not including all eligible patients in their analyses. All but one study scored a high risk of bias in the analysis domain, commonly due to studies removing patients with missing data from all their analyses, not adjusting the developed model for under or overfitting, or not accounting for complexities in the data, such as censoring.

## Discussion

### Principal Findings

This systematic review builds on our recent review on the association between blood test trend and cancer diagnosis [13] by highlighting the potential for risk stratification and methodological considerations of incorporating combinations of trends into cancer risk prediction models for use in practice. Our review identified logistic regression (incorporating the difference between 2 blood tests as a single variable) and multivariate joint modeling as the most commonly used modeling techniques. Models were often developed using poor methods. For example, although all but one model underwent internal validation during model development, model performance was not adequately assessed, with calibration often ignored and recalibration rarely performed for overfitting [37-41]. Where calibration was assessed, the Hosmer-Lemeshow test was sometimes used, which is known to have limited power and poor interpretability [37]. Many models were inadequately reported, with only one study providing the full risk-equation needed to derive an individual’s risk of diagnosis. Without the full risk equation being available, models are unlikely to be independently externally validated or easily embedded into practice. Although our primary focus was to critically appraise trend-based prediction models, it is important to also highlight caution in the interpretation of performance measures from the models, as these may be subject to publication bias. For example, a prediction model with a poorer c-statistic is less likely to be published.

The ColonFlag model was most commonly externally validated, although this model is commercially developed so not publicly available. This model uses trends in FBC parameters to predict a monotonic score confined between 0‐100, where higher scores reflect a higher likelihood of colorectal cancer diagnosis [27]. A pooled c-statistic of 0.81 from 4 studies indicates that trends in the FBC could be generalizable to other clinical settings and geographical locations, with good predictive ability to distinguish between patients with and without colorectal cancer. Heterogeneity was however high. This was anticipated due to the variation between studies included in the meta-analysis, such as differing geographical settings, health care systems, and eHealth records used. Therefore, caution should be given in the interpretation of these results when making generalisations between different clinical settings. There were few studies demonstrating the external validity of other models including blood test trend. Predictive ability of models was not assessed by cancer characteristics, such as by cancer stage, in any study.

### Comparison of Models

A total of 3 models were identified for colorectal cancer: the ColonFlag and sex-specific BLOODTRACC models. Both models include age and sex, with the ColonFlag also including trend in all 20 FBC parameters and the BLOODTRACC models including trend in only three FBC parameters (hemoglobin, mean cell volume, and platelets). The ColonFlag uses changes over tests measured at 36 and 18 months up to the current test, with all patients requiring a test at each time point, whereas the BLOODTRACC models use all available tests over a five-year period before the current test and takes into consideration the timing of tests, as blood tests are not performed routinely in the United Kingdom. Although the ColonFlag was developed for 3‐6 month risk in Israeli primary care, external validation studies of this model for two-year risk found it performed similarly to the BLOODTRACC models for 2-year risk in UK primary care. This suggests that the 17 additional blood test trends in the ColonFlag may not add further diagnostic benefit to the combination of hemoglobin, mean corpuscular volume, and platelet trends for colorectal cancer. This may suggest that the underlying methodology used to develop the models (decision trees for the ColonFlag and joint modeling for the BLOODTRACC models) does not affect discriminative performance, but this would need assessing on the same patient dataset and multiple study designs employed to reduce heterogeneity. This assessment was performed in the BLOODTRACC model derivation study, where both models derived comparable c-statistics in the same cohort, both overall and in subgroups of age, by number of blood tests used to derive trends, and by longitudinal period used to derive trends [31].

Read et al[28] developed two models for gastro-intestinal cancer, one using random forests and one using logistic regression. Both models were designed to be as similar as possible, such as using the same study sample, outcome window, longitudinal period to derive trends, and similar covariates, with the methodological approach used to derive the methods being the biggest difference. Both models achieved an AUC of 0.71, suggesting that the underlying methodological approach may not affect discriminative performance, although the logistic model had better overall performance (lower Brier score). Neither model was assessed for calibration so further testing is required.

The remaining 2 models were for lung and pancreatic cancer. These were not compared with other models, as no further models for lung or pancreatic cancer were identified.

### Strengths and Limitations

To our knowledge, this is the first review of cancer prediction models that incorporate blood test trend. We performed a comprehensive search, developed with an information specialist, including full-length articles retrieved from MEDLINE and EMBASE. It is possible that additional relevant studies may be found exclusively in other databases and were missed by our review. However, it is likely that most relevant manuscripts were found, as MEDLINE and EMBASE had 97.5% coverage of articles in previous systematic reviews and we conducted citation searching of all included manuscripts [42]. Our review identified prediction models for only four cancer types, with two externally validated (colorectal and pancreatic). We were therefore unable to draw conclusions regarding external validity for many cancer types. One further limitation is that we were unable to draw conclusions regarding publication bias, assessing whether prediction models were more likely to be published if they had good predictive performance. Only five models had c-statistics with corresponding confidence intervals at internal validation, making it difficult to assess symmetry in a funnel plot and deduce any publication bias.

### Comparison With Previous Work

To date, prediction models for cancer risk are most commonly developed using single blood test results (plus other predictors). These include the QCancer models for the 2-year risk of cancer [43,44] and unexpected weight loss models for the 6-month risk of cancer [45], which combine patient demographics, symptoms, and single blood test values for cancer risk in symptomatic patients in UK primary care practices. Collectively, these models have c-statistics ranging 0.79‐0.92, comparable to 0.71‐0.87 reported for the models included in this review, which often included only blood test trends, age, and sex and different outcome risk windows. Existing systematic reviews have identified prediction models for individual cancer sites, including lung, breast, colorectal, and prostate, but the focus of these reviews was not on the role of blood test trend [46-49]. Lung cancer prediction models in those reviews often included patient demographics, pneumonia, exposure to smoking, and single blood tests for one-year risk, with c-statistic ranging 0.66‐0.91. In this review, Gould et al [23] reported 0.87 for six-month risk of lung cancer using similar predictors combined with trend in seven blood tests. Colorectal cancer prediction models in those reviews often included patient demographics and single blood tests, with c-statistic ranging from 0.82‐0.84 for 6-month risk and 0.72‐0.92 for 2-year risk. In this review, Kinar et al [27] and Birks et al [21] reported 0.82‐0.84 for 6-month risk and Virdee et al [31] reported 0.75‐0.76 for 2-year risk of colorectal cancer using trend in 20 and three blood tests, respectively, age, and sex. Although those reviews identified prediction models using single blood test results for breast and prostate cancer [46,49], we found no prediction models incorporating trends for these cancers in this systematic review.

### Clinical and Research Implications

Thorough testing of prediction models is required before clinical guidelines for cancer investigation can incorporate blood test trends. This includes assessment for the predictive ability of blood test trend compared to single blood tests and symptoms and the potential for early detection of cancer. For example, in the cancer field, the NICE guidelines recommend primary care to refer for cancer investigation if a patient’s risk is above 3%, which is often used to support referral of symptomatic patients, whose risk is likely higher than nonsymptomatic patients. For models derived for more general populations, such as the trend-based models included in this review, there is no clear cut-off. To assess the potential added benefit of trend, studies would need to compare the diagnostic accuracy of trend-based and static/single-test models. No study in our review performed such comparisons, so this potential remains unknown. Patient- and clinician-acceptability of blood test trend approaches for cancer detection also requires investigation to optimize uptake of such models in practice. As some clinicians order blood tests more than others, methods to standardize blood testing across practices may be warranted and could reduce practice-level variability through clinical guidelines on repeat blood testing. This additional testing may add burden to health care, but the balance of patient benefit and outcomes to health care burden would need investigation. In terms of reporting, prediction models were often not reported in full, which is required for implementation into clinical systems and use in practice. Future models should follow appropriate reporting guidelines to ensure they are appropriately reported, such as the TRIPOD [36] or TRIPOD-AI [50] guidelines.

Sub-optimal methods to analyse trends were often identified, such as logistic regression incorporating change between tests. Recent technological advancements have allowed for dynamic models, which are designed for repeated measures data by appropriately accounting for nonindependent data sporadically recorded in routine clinical practice [51], to be incorporated into analysis software packages. These include models such as landmarking and joint modeling of longitudinal and time-to-event data [52-54]. Research is required to assess the implementation considerations of different methodological techniques. For example, the feasibility of incorporating computationally intensive approaches, such as joint modeling, or approaches that require larger datasets or are nontransparent, such as machine learning. Our ongoing research aims to develop and validate trend-based prediction models for cancer, with eventual integration of trend into risk stratification in clinical practice [55]. Future prediction model studies should employ appropriate validation metrics, as we found that most studies did not assess overall performance or calibration. Further sub-optimal analysis methods commonly used included removing patients with missing data from all their analyses, not adjusting the developed model for under or overfitting, or not accounting for complexities in the data, such as censoring. Future models should consider such points to reduce bias.

### Conclusion

We highlight the cancers for which there is a reported prediction model incorporating changes in repeated blood tests over time and the cancers and blood tests with no published literature. We provide an overview of the predictive performance of prediction models incorporating blood test trends and highlight that further testing is needed for all models identified. This review lays the foundation for further research.

## Supplementary material

10.2196/70275 Multimedia Appendix 1 Final search strategy.

10.2196/70275 Checklist 1 PRISMA checklist.

## Abbreviations

ENDPAC: Enriching New-Onset Diabetes for Pancreatic Cancer
FBC: full blood count
ICD10: International Statistical Classification of Diseases and Related Health Problems 10th Revision
NICE: National Institute for Health and Care Excellence
PRISMA: Preferred Reporting Items for Systematic review and Meta-Analysis
PROBAST: prediction model risk of bias assessment tool
PROSPERO: Prospective Register of Systematic Reviews
TRIPOD: Transparent Reporting of a multivariable prediction model for Individual Prognosis Or Diagnosis
