# Supplementary material for: Clinical Prediction Models Incorporating Blood Test Trend for Cancer Detection: Systematic Review, Meta-Analysis, and Critical Appraisal
Source: JMIR Cancer. 2025 Jun 27;11:e70275. doi: 10.2196/70275 (PMC12227175; doi:10.2196/70275)
Supplement: Multimedia Appendix 1 [file cancer-v11-e70275-s001.docx]

Table S1: Final search strategy (MEDLINE)

| **MEDLINE** |
| --- |
| Database and platform: Medline (Ovid MEDLINE® Epub Ahead of Print, In-Process & Other Non-Indexed Citations, Ovid MEDLINE® Daily and Ovid MEDLINE®) 1946 to present. Search date: 3^rd^ April 2025.  1 exp Neoplasms/bl [Blood]  2 exp Neoplasms/  3 (neoplas* or tumor* or tumour* or cancer* or malignan* or carcino* or sarcom* or leukaem* or leukem* or lymphom* or melano* or metasta* or mesothelio* or mesotelio* or carcinomatos* or gliom* or glioblastom* or osteosarcom* or blastom* or neuroblastom* or oncolog* or myelodysplas* or adenocarcinoma* or choriocarcinoma*).ti.  4 2 or 3  5 Hematologic Tests/  6 exp Blood Cell Count/  7 exp Blood Cells/an  8 Blood Sedimentation/  9 Blood Viscosity/  10 exp Hemoglobins/an, bl  11 Hematocrit/  12 Erythrocyte Indices/ or Erythrocytes/an, bl  13 platelet function tests/ or mean platelet volume/  14 Liver Function Tests/  15 alanine transaminase/an, bl or exp aspartate aminotransferases/an, bl  16 Albumins/an or Albuminuria/bl, ur  17 serum albumin/ or serum albumin, human/  18 exp Bilirubin/an, bl  19 alpha-Fetoproteins/an, bl  20 Alkaline Phosphatase/an, bl [Analysis, Blood]  21 exp Kidney Function Tests/  22 Sodium/an, bl, ur  23 Potassium/an, bl, ur  24 Creatinine/an, bl, ur  25 Urea/an, bl, ur  26 Amylases/an, bl  27 Calcium/an, bl, ur  28 Glycated Hemoglobin A/an, bl or Blood Glucose/an, bl  29 Blood Proteins/an, bl  30 C-Reactive Protein/an, bl  31 exp Thyrotropin/an, bl  32 ((hemoglobin? or haemoglobin? or hb) adj3 (variation? or level? or concentration? or declin* or mean cell)).ti,ab,kf. or (hemoglobin? or haemoglobin?).ti.  33 ((complete blood or whole blood or blood cell or white cell or erythrocyte? or leukocyte? or platelet? or lymphocyte? or eosinophil? or neutrophil? or basophil? or monocyte?) adj2 (count? or variation?)).ti,ab,kf.  34 ((lymphocyte? or eosinophil? or neutrophil? or basophil? or monocyte?) adj2 (percent* or "%")).ti,ab,kf.  35 (erythrocyte sedimentation or blood sedimentation or blood viscosity or mean platelet or mean cell volume or hematocrit? or haematocrit? or ((blood cell or erythrocyte?) adj2 (index or indices or distribution))).ti,ab,kf.  36 ((c-reactive protein or crp) adj3 (plasma or serum or blood or variation? or level? or concentration? or elevat? or increas* or high*)).ti,ab,kf. or (c-reactive protein or crp).ti.  37 ((total or serum or blood or plasma) adj2 protein?).ti,ab,kf. or protein.ti.  38 ((albumin? adj3 (plasma or serum or blood or variation? or level? or concentration? or declin*)) or albumin creatinine ratio?).ti,ab,kf. or albumin*.ti.  39 ((alkaline phosphatase or alp) adj3 (plasma or serum or blood or variation? or level? or concentration? or elevat? or increas* or high*)).ti,ab,kf. or (alkaline phosphatase or alp).ti.  40 ((aminotransferase or ast or sgot or alt or sgpt) adj3 (plasma or serum or blood or variation? or level? or concentration? or elevat? or increas* or high*)).ti,ab,kf. or (aminotransferase or ast or sgot or alt or sgpt).ti.  41 (bilirubin adj3 (plasma or serum or blood or variation? or level? or concentration? or elevat? or increas* or high*)).ti,ab,kf. or bilirubin.ti.  42 (liver enzyme? adj3 (plasma or serum or blood or variation? or level? or concentration? or elevat? or increas* or high*)).ti,ab,kf. or (liver enzyme? or liver function).ti. or liver function test*.ti,ab,kf.  43 (renal funtion test* or kidney function test*).ti,ab,kf. or (renal function or kidney function).ti.  44 (sodium adj3 (plasma or serum or blood or variation? or level? or concentration? or declin*)).ti,ab,kf. or sodium.ti.  45 (potassium adj3 (plasma or serum or blood or variation? or level? or concentration? or elevat? or increas* or high*)).ti,ab,kf. or potassium.ti.  46 (creatinine adj3 (plasma or serum or blood or variation? or level? or concentration? or elevat? or increas* or high*)).ti,ab,kf. or creatinine.ti.  47 ((urea adj3 (plasma or serum or blood or variation? or level? or concentration? or decline?)) or urea cycle?).ti,ab,kf. or urea.ti.  48 (amylase adj3 (plasma or serum or blood or variation? or level? or concentration? or elevat? or increas* or high*)).ti,ab,kf. or amylase.ti.  49 (((glucose or hba1c) adj3 (plasma or serum or blood or variation? or level? or concentration? or elevat? or increas* or high*)) or fasting glucose).ti,ab,kf. or (glucose or hba1c).ti.  50 (calcium adj3 (plasma or serum or blood or variation? or level? or concentration? or elevat? or increas* or high*)).ti,ab,kf. or calcium.ti. or calcium adjusted.ti,ab,kf.  51 (((thyrotropin? or thyroid stimulating hormone?) adj3 (plasma or serum or blood or variation? or level? or concentration? or elevat? or increas* or high*)) or fasting glucose).ti,ab,kf. or (thyrotropon? or thyroid stimulating hormone?).ti.  52 anemia/ or anemia, hypochromic/ or anemia, iron-deficiency/ or exp anemia, macrocytic/  53 (an?emia? or an?emic or microcytosis or microcytic).ti,ab,kf.  54 or/5-53  55 4 and 54  56 1 or 55  57 exp Neoplasms/di  58 early diagnosis/ or "early detection of cancer"/  59 (detect* or diagnos* or screen*).ti.  60 ((neoplas* or tumor* or tumour* or cancer* or malignan* or carcino* or sarcom* or leukaem* or leukem* or lymphom* or melano* or metasta* or mesothelio* or mesotelio* or carcinomatos* or gliom* or glioblastom* or osteosarcom* or blastom* or neuroblastom* or oncolog* or myelodysplas* or adenocarcinoma* or choriocarcinoma*) adj3 (diagnos* or detect* or screen*)).ti,ab,kf.  61 57 or 58 or 59 or 60  62 56 and 61  63 (trend? or pattern? or lead time* or timeline* or time line* or time frame? or time frame? or interval?).ti,ab,kf.  64 ((longterm or long-term) adj2 (variation? or chang* or difference* or declin* or decreas* or increas* or elevat* or level? or concentration)).ti,ab,kf.  65 (prediagnos* or pre-diagnos* or ((before or prior) adj2 diagnos*)).ti,ab,kf.  66 ((risk? or predict*) adj5 (model* or logarithm* or algorithm* or machine learning)).ti,ab,kf.  67 (model* or logarithm* or algorithm* or machine learning).ti.  68 (risk? adj2 (scor* or model* or index or indices or tool* or assessment? or measurement?)).ti,ab,kf.  69 (Models, Biological/ or Models, Statistical/) and exp Risk/  70 63 or 64 or 65 or 66 or 67 or 68 or 69  71 62 and 70  72 exp animals/ not humans/  73 71 not 72 |

Table S2: Final search strategy (EMBASE)

| **EMBASE** |
| --- |
| Database and platform: Embase 1974 to present. Search date: 3^rd^ April 2025.  1 exp *neoplasm/  2 (neoplas* or tumor* or tumour* or cancer* or malignan* or carcino* or sarcom* or leukaem* or leukem* or lymphom* or melano* or metasta* or mesothelio* or mesotelio* or carcinomatos* or gliom* or glioblastom* or osteosarcom* or blastom* or neuroblastom* or oncolog* or myelodysplas* or adenocarcinoma* or choriocarcinoma*).ti.  3 1 or 2  4 hematological parameters/  5 exp blood cell count/  6 exp blood cell/an  7 erythrocyte sedimentation rate/  8 Blood Viscosity/  9 exp hemoglobin/an  10 hemoglobin blood level/ or "hemoglobin determination"/  11 Hematocrit/  12 exp erythrocyte parameters/  13 blood clotting parameters/ or exp platelet volume/  14 exp liver function test/  15 enzyme blood level/  16 alanine aminotransferase blood level/ or aminotransferase blood level/ or aspartate aminotransferase blood level/  17 protein blood level/  18 albumin blood level/  19 bilirubin blood level/  20 alpha fetoprotein blood level/  21 alkaline phosphatase blood level/  22 exp kidney function test/ or electrolyte blood level/  23 sodium blood level/  24 potassium blood level/  25 creatine kinase blood level/ or creatinine blood level/  26 urea blood level/  27 amylase blood level/  28 calcium blood level/  29 glucose blood level/ or exp *hemoglobin A1c/  30 C reactive protein/an  31 exp thyroid hormone blood level/ or thyrotropin blood level/  32 ((hemoglobin? or haemoglobin? or hb) adj3 (variation? or level? or concentration? or declin* or mean cell)).ti,ab,kf. or (hemoglobin? or haemoglobin?).ti.  33 ((complete blood or whole blood or blood cell or white cell or erythrocyte? or leukocyte? or platelet? or lymphocyte? or eosinophil? or neutrophil? or basophil? or monocyte?) adj2 (count? or variation?)).ti,ab,kf.  34 ((lymphocyte? or eosinophil? or neutrophil? or basophil? or monocyte?) adj2 (percent* or "%")).ti,ab,kf.  35 (erythrocyte sedimentation or blood sedimentation or blood viscosity or mean platelet or mean cell volume or hematocrit? or haematocrit? or ((blood cell or erythrocyte?) adj2 (index or indices or distribution))).ti,ab,kf.  36 ((c-reactive protein or crp) adj3 (plasma or serum or blood or variation? or level? or concentration? or elevat? or increas* or high*)).ti,ab,kf. or (c-reactive protein or crp).ti.  37 ((total or serum or blood or plasma) adj2 protein?).ti,ab,kf. or protein.ti.  38 ((albumin? adj3 (plasma or serum or blood or variation? or level? or concentration? or declin*)) or albumin creatinine ratio?).ti,ab,kf. or albumin*.ti.  39 ((alkaline phosphatase or alp) adj3 (plasma or serum or blood or variation? or level? or concentration? or elevat? or increas* or high*)).ti,ab,kf. or (alkaline phosphatase or alp).ti.  40 ((aminotransferase or ast or sgot or alt or sgpt) adj3 (plasma or serum or blood or variation? or level? or concentration? or elevat? or increas* or high*)).ti,ab,kf. or (aminotransferase or ast or sgot or alt or sgpt).ti.  41 (bilirubin adj3 (plasma or serum or blood or variation? or level? or concentration? or elevat? or increas* or high*)).ti,ab,kf. or bilirubin.ti.  42 (liver enzyme? adj3 (plasma or serum or blood or variation? or level? or concentration? or elevat? or increas* or high*)).ti,ab,kf. or (liver enzyme? or liver function).ti. or liver function test*.ti,ab,kf.  43 (renal funtion test* or kidney function test*).ti,ab,kf. or (renal function or kidney function).ti.  44 (sodium adj3 (plasma or serum or blood or variation? or level? or concentration? or declin*)).ti,ab,kf. or sodium.ti.  45 (potassium adj3 (plasma or serum or blood or variation? or level? or concentration? or elevat? or increas* or high*)).ti,ab,kf. or potassium.ti.  46 (creatinine adj3 (plasma or serum or blood or variation? or level? or concentration? or elevat? or increas* or high*)).ti,ab,kf. or creatinine.ti.  47 ((urea adj3 (plasma or serum or blood or variation? or level? or concentration? or decline?)) or urea cycle?).ti,ab,kf. or urea.ti.  48 (amylase adj3 (plasma or serum or blood or variation? or level? or concentration? or elevat? or increas* or high*)).ti,ab,kf. or amylase.ti.  49 (((glucose or hba1c) adj3 (plasma or serum or blood or variation? or level? or concentration? or elevat? or increas* or high*)) or fasting glucose).ti,ab,kf. or (glucose or hba1c).ti.  50 (calcium adj3 (plasma or serum or blood or variation? or level? or concentration? or elevat? or increas* or high*)).ti,ab,kf. or calcium.ti. or calcium adjusted.ti,ab,kf.  51 (((thyrotropin? or thyroid stimulating hormone?) adj3 (plasma or serum or blood or variation? or level? or concentration? or elevat? or increas* or high*)) or fasting glucose).ti,ab,kf. or (thyrotropon? or thyroid stimulating hormone?).ti.  52 anemia/ or exp iron deficiency anemia/ or exp macrocytic anemia/  53 (an?emia? or an?emic or microcytosis or microcytic).ti,ab,kf.  54 or/4-53  55 exp Neoplasm/di  56 early diagnosis/ or cancer diagnosis/ or early cancer diagnosis/  57 (detect* or diagnos* or screen*).ti.  58 ((neoplas* or tumor* or tumour* or cancer* or malignan* or carcino* or sarcom* or leukaem* or leukem* or lymphom* or melano* or metasta* or mesothelio* or mesotelio* or carcinomatos* or gliom* or glioblastom* or osteosarcom* or blastom* or neuroblastom* or oncolog* or myelodysplas* or adenocarcinoma* or choriocarcinoma*) adj3 (diagnos* or detect* or screen*)).ti,ab,kf.  59 55 or 56 or 57 or 58  60 (trend? or pattern? or lead time* or timeline* or time line* or time frame? or time frame? or interval?).ti,ab,kf.  61 ((longterm or long-term) adj2 (variation? or chang* or difference* or declin* or decreas* or increas* or elevat* or level? or concentration)).ti,ab,kf.  62 (prediagnos* or pre-diagnos* or ((before or prior) adj2 diagnos*)).ti,ab,kf.  63 ((risk? or predict*) adj5 (model* or logarithm* or algorithm* or machine learning)).ti,ab,kf.  64 (model* or logarithm* or algorithm* or machine learning).ti.  65 (risk? adj2 (scor* or model* or index or indices or tool* or assessment? or measurement?)).ti,ab,kf.  66 (statistical model/ or biological model/ or disease model/ or cancer model/) and (risk/ or *risk assessment/)  67 60 or 61 or 62 or 63 or 64 or 65 or 66  68 3 and 54 and 59 and 67  69 exp animal/ not human/  70 68 not 69  71 conference*.pt.  72 70 not 71  73 70 and 71 |

## Table S3: Detailed description of the 16 studies included in the review

| **Article** | **Development or validation or both** | **Study type** | **Study design** | **Country** | **Patient setting** | **Cancer** | **Restricted to symptomatic patients?** | **Reason for blood testing^1^** | **Data source** | **Patient population** | **Average age^2^** | **% female** | **No. blood tests** | **Average time from first test to diagnosis / censor** |
| --- | --- | --- | --- | --- | --- | --- | --- | --- | --- | --- | --- | --- | --- | --- |
| Ayling 2019 | Validation | Prospective | Cohort | UK | Secondary care | Colorectal | No | Opportunistic tests | Clinical centres | Patients seen in the Gastroenterology Clinic in Derriford Hospital, Plymouth, between March 2014 and March 2017, were invited to participate. They had been referred from Primary Care with a low haemoglobin concentration, ostensibly secondary to iron deficiency, on a 2-week wait cancer pathway. Additionally, a cohort of consecutive iron-deficient patients who attended the Royal London Hospital for colonoscopy during 2017. |  |  |  |  |
| Ayling 2021 | Validation | Prospective | Cohort | UK | Secondary care | Colorectal | No | Opportunistic tests | Clinical centres | All adult patients over 40 years of age, referred to Barts Health NHS Trust on an urgent pathway with suspected CRC and awaiting investigation on 1 May 2020 were included in the study. | 63 |  | 8 |  |
| Birks 2017 | Validation | Retrospective | Cohort | UK | Primary care | Colorectal | No | Opportunistic tests | Electronic health records: CPRD GOLD | All patients included in the study had at least one FBC present in the record. Entry to the cohort was at the index date, which is defined as the latest of start date, the 40th birthday, or date of registration with the general practice. We excluded the following groups: • Less than 12 months registered with the general practice • Less than 2 years of follow-up data following the index date • History of colorectal cancer before the index date • Colorectal cancer precursors (e.g., adenomatous polyps) • Hemoglobin gene defects (thalassemia, sickle cell disorders) | 54.2 | 55.4 |  | 18-24 months |
| Boursi 2022 | Validation | Retrospective | Cohort | Israel | Primary care | Pancreatic | Anyone | Opportunistic tests | Electronic health records: Maccabi Health Services; Israel Cancer Registry | Study participants included patients 50 years or older that had a physician diagnosis of new onset diabetes (NOD) at the MHS diabetes registry between 2006 and 2015. Eligible patients were required to have (1) fasting plasma glucose (FPG) or glycated hemoglobin (HbA1c) measurement at index date and 3 to 18 months before index date, (2) body weight measurement at the index date and 3 to 18 months before index date. Additional exclusion criteria included patients with FPG less than 126 mg/dL at index date or FPG greater than 126 mg/dL at the pre-index date period, and subjects with PDAC before the initial diagnosis of NOD. |  | 49.5 |  | 3-18 months |
| Chen 2024 | Validation | Retrospective | Cohort | USA | Other | Pancreatic | Anyone | Opportunistic tests | Electronic health records: Kaiser Permanente Southern California | Patients had an elevated HbA1c. 1 non-elevated HbA1c or fasting glucose measure in the window was required. No evidence of prior diabetes mellitus (DM). No history of pancreatic cancer. Continuously enrolled in the KPSC health plan on the index date and in the next three years. Patients who died of non-pancreatic cancer within 3 years after the index date were excluded. At least one body weight measure around the index date and in the time interval of 6-18 months. In addition, at least one HbA1c measure in the time interval of 6-18 months. |  |  |  | 6-18 months |
| Goshen 2018 | Validation | Prospective | Cohort | Israel | Unclear | Colorectal | No | Opportunistic tests | Electronic health records: Maccabi Health Maintenance Organization | The study group consisted of individuals who had a CBC result reported within the study period and who were not compliant with standard screening recommendations; that is, they did not have a screening colonoscopy in the past 10 years and did not have a FIT screening test done in the 18-month period before the date of the index CBC (index date). We also excluded individuals who had been referred for a FIT screening test in the past 3 months but who had not yet completed the FIT test. Individuals were also excluded if they had previously been diagnosed with any cancer. |  |  |  |  |
| Gould 2021 | Development | Retrospective | Case-control | USA | Other – insured individuals | Non-small cell lung cancer | No | Opportunistic tests | Electronic health records: Kaiser Permanente Southern California | We identified case patients and control subjects by using data from the KPSC Cancer Registry and Research Data Warehouse. Case patients included KPSC members with lung cancer diagnosed between 2008 and 2015 who were 45–90 years old on the date of diagnosis. Control subjects included KPSC members without a current or prior diagnosis of lung cancer who were 45–90 years old on an assigned index (pseudodiagnosis) date of July 1. On the basis of the findings of prior research, we required the presence of at least one CBC in the 12 months before the indexed date of diagnosis (for case patients) or pseudodiagnosis (for control subjects). We excluded case patients and control subjects who had not been continuously enrolled for at least 12 months before the most recent outpatient CBC preceding the index date. To assemble the final set of control subjects without lung cancer, we randomly sampled (without replacement) 5% of nonexcluded individuals on each index date. |  |  |  | 0-12 months |
| Hilsden 2018 | Validation | Prospective | Cohort | Canada | Other – screening unit | Colorectal | No: asymptomatic only | Opportunistic tests | Clinical centres and electronic health records: Alberta Health Services’ Analytics, Alberta Cancer Registry | Individuals between the ages of 50 and 75 who underwent a colonoscopy at the Centre between January 2013 and June 2015. To be included in the study, the patient must have undergone a successful colonoscopy (complete to the cecum unless incomplete due to an obstructing mass) with a bowel preparation rated by the endoscopist as adequate to detect polyps greater than 5 mm in size. Three subgroups of patients were eligible for the study (1) individuals at average risk for colorectal cancer, (2) individuals with a personal history of polyps and (3) individuals with a family history of polyps or colorectal cancer. Patients were excluded if they had a positive guaiac or immunochemical fecal occult blood test, a prior history of colorectal cancer, a known or suspected genetic predisposition to cancer or no CBC result within the year prior to their colonoscopy. | 50-69 |  |  |  |
| Hornbrook 2017 | Validation | Retrospective | Case-control | USA | Other – insured individuals | Colorectal | No | Opportunistic tests | Electronic health records: Kaiser Permanente Northwest Region; Kaiser Permanente Tumor Registry | The colorectal cancer cases were selected from the KP Tumor Registry using the following selection criteria: (1) diagnosed with colorectal cancer; (2) had one or more CBCs within 6 months of the CRC diagnosis date; (3) had at least 180 days of continuous KPNW enrollment prior to CRC diagnosis date; (4) CRC patients with any cancer diagnosis prior to the CRC diagnosis date were excluded; and (5) CRC patients with other cancers diagnosed on the same date as the CRC diagnosis date were flagged so that this variable was available to the detection modeling effort. Control cases were selected from the KPNW membership using the following criteria: (1) received at least one outpatient CBC between 2000 and 2013; (2) age between age 40 and 89 years at time of at least one CBC; (3) no history of cancer diagnoses in the KPNW Tumor Registry or electronic medical record systems; (4) were continuously enrolled in KPNW from 180 days prior to CBC date through 24 months after the CBC date (30 months of cancer-free continuous enrollment, with gaps of up to 3 months patched). A random sample of 900 KPNW adults with CRC (and having at least one prior CBC) who were at least 40 years of age at the time of disease onset, and a random sample of 16,195 healthy KPNW controls were created. |  | 55.8 |  | 0-6 months |
| Khan 2021 | Validation | Retrospective | Cohort | USA | Unclear | Pancreatic | Anyone | Opportunistic tests | Electronic health records: TrinetX | Patients were selected who met the glycemic definition of new-onset diabetes. An HbA1c > 6.5 was preceded by at least one HbA1c < 6.5 in the past 6-24 months. The date the HbA1c > 6.5 was obtained was defined as the index date. No HbA1c > 6.5 before the index date, exposure to anti-diabetic medications occurred up until three months before the index date, history of pancreatic cancer before the index date, or age less than 50 years at index date. |  |  |  |  |
| Kinar 2016 | Both | Retrospective | Cohort | Israel | Primary care | Colorectal | No | Opportunistic tests | Electronic health records: Maccabi Healthcare Services; Israeli National Cancer Registry | All insured individuals aged 50-75 years. Individuals diagnosed with cancer other than CRC were excluded. |  | 53.5 |  | 39-42 months |
| Kinar 2017 | Validation | Retrospective | Cohort | Israel | Primary care | Colorectal | No | Opportunistic tests | Electronic health records: Maccabi Healthcare Services; Israeli National Cancer Registry | All men and women between ages 50 and 75 on January 1, 2008 who had one or more CBC report recorded in the MHS electronic medical record system for a blood sample taken during the six month testing period. This CBC report was called the index report. Subjects were excluded if they had a diagnosis of CRC or any other cancer recorded in the National Cancer Registry prior to January 1, 2008 or if they had no index blood test taken during the testing period. | 60.9 |  |  | 12-18 months |
| Read 2023 | Development | Retrospective | Cohort | USA | Primary care | GI | No | Opportunistic tests | Clinical centres | Subjects were identified as individuals from the Michigan Medicine Clinical Data Warehouse who had at least 2 CBCs within a rolling 2-year time frame between 1 January 2004 and 31 December 2013. Michigan Medicine is a large referral center as well as a primary care system. We used the presence of 2 CBCs to identify patients seeking regular care at Michigan Medicine. Subjects were excluded if age < 18, given the low incidence of GI tract cancers and paucity of routine blood draws in a pediatric population. | 49.4 | 62.1 | 2+ |  |
| Schneider 2020 | Validation | Retrospective | Case-control | USA | Other – insured individuals | Colorectal | No | Opportunistic tests | Electronic health records: Kaiser Permanente Northwest Region | KPNC Health Plan members between 1996 and 2015 who had at least 1 outpatient CBC test for any indication with at least 3 years of prior CBC data. The primary analyses for the current study used a 40% random subset of the study population, which was restricted to patients at least 50 years of age. Cases were selected from eligible cohort members who received a CBC between 50 and 75 years of age, did not have a prior or current CRC diagnosis by the CBC date, and were subsequently diagnosed with CRC. Controls were individuals between 50 and 75 years of age at the date of a randomly selected blood count test with no CRC diagnosis. |  | 52 |  |  |
| Sharma 2018 | Both | Retrospective | Cohort | USA | Unclear | Pancreatic | No | Opportunistic tests | Electronic health records: Rochester Epidemiology Project | All new-onset diabetes subjects in Olmsted County between January 1st, 2000 to December 31st, 2015 (n=1561) were identified using a glycemic definition of diabetes (Supplementary material, Table 1). Among these subjects, 1288 (83%) had available the prior data on weight and blood glucose between 3 and 18 months prior as well as when first meeting new-onset diabetes criteria (paired data). |  |  |  | 39-54 months |
| Virdee 2022 | Both | Retrospective | Cohort | UK | Primary care | Colorectal | No | Opportunistic tests | Electronic health records: CPRD; NCRAS | Patients aged at least 40 years with at least one haemoglobin, mean corpuscular volume (MCV), and platelet measurement available in their primary care record were included. Patients were excluded if registered with their primary care practice for less than one year, had a history of colorectal cancer before study entry, or not linked to the NCRAS registry. Patients with an available date of diagnosis but no indication of cancer were excluded. For the internal validation cohort, patients with no ColonFlag score corresponding to the baseline FBC were excluded to ensure the models were compared on the same patient sample and two-year risk scores from the same FBC. The ColonFlag score was derived by Medial EarlySign and returned to us for analysis, with reasons for missing ColonFlag scores unknown. | 61.31 | 55.9 | 2-4 | Males: 42-48 months  Females: 48-54 months |

^1^Opportunistic tests are those performed for various reasons but are being utilised for cancer assessment.

^2^Case-control studies: mean age at index date (diagnosis for cases, censor for controls). Other studies: mean age at study entry.

## Table S4: Blood test trends details and model type per study

| **Article** | **Model type** | **Max longitudinal period for blood testing** | **Nature of repeat blood testing** | **If regularly taken, how often** | **Average no. blood tests used for trend** | **No. blood tests used in total** |
| --- | --- | --- | --- | --- | --- | --- |
| Gould 2021 | XGBoost | 5 years | Sporadic |  |  |  |
| Kinar 2016 | Decision trees | 3 years | Regular intervals | 18 months |  |  |
| Read 2023 | Logistic regression | 3 years | Sporadic |  |  |  |
| Read 2023 | Random forest | 3 years | Sporadic |  | 2+ |  |
| Sharma 2018 | Logistic regression | 18 months | Other |  | 2+ | 3122 |
| Virdee 2022 | Multivariate joint models (females) | 5 years | Sporadic |  | Cases=3, non-cases=3 | 913142 |
| Virdee 2022 | Multivariate joint models (males) | 5 years | Sporadic |  | Cases=3, non-cases=2 | 805164 |

Figure S1: Forest plot of c-statistic for risk of colorectal cancer from ColonFlag external validations: excluding USA (top) and USA-only (bottom) studies

## Table S5: Risk of bias in the 16 studies developing/validating trend-based prediction models, assessed using the PROBAST tool

| **Article** | **Study type** | **Model (if assigned)** | **Participants** | **Predictors** | **Outcome** | **Analysis** |
| --- | --- | --- | --- | --- | --- | --- |
| Ayling 2019 | External validation | ColonFlag | Low | Low | Low | High |
| Ayling 2021 | External validation | ColonFlag | Low | Low | Low | High |
| Birks 2017 | External validation | ColonFlag | Low | Low | Low | High |
| Boursi 2022 | External validation | ENDPAC | Low | Low | Low | High |
| Chen 2024 | External validation | ENDPAC | Low | Low | Low | High |
| Goshen 2018 | External validation | ColonFlag | Low | Low | Low | High |
| Gould 2021 | Development | MES | High | Low | Low | High |
| Hilsden 2018 | External validation | ColonFlag | Low | Low | Low | High |
| Hornbrook 2017 | External validation | ColonFlag | High | Low | Low | High |
| Khan 2021 | External validation | ENDPAC | Low | Low | Low | High |
| Kinar 2016 | Both | ColonFlag | Low | Low | Low | High |
| Kinar 2017 | External validation | ColonFlag | Low | Low | Low | High |
| Read 2023 | Development |  | Low | Low | Low | High |
| Schneider 2020 | External validation | ColonFlag | High | Low | Low | High |
| Sharma 2018 | Both | ENDPAC | Low | Low | Low | High |
| Virdee 2022 | Both | Developed: BLOODTRACC Colorectal; Externally validated: ColonFlag | Low | Low | Low | Low |
| Total low (%) |  |  | 13 (81%) | 16 (100%) | 16 (100%) | 1 (6%) |
| Total high (%) |  |  | 3 (19%) | 0 | 0 | 15 (94%) |
| Total unclear (%) |  |  | 0 | 0 | 0 | 0 |
